# Donanemab therapy in Alzheimer’s disease with mild cognitive impairment: Convergent amyloid, tau, and plasma biomarker normalization with cognitive improvement

**DOI:** 10.1016/j.tjpad.2026.100533

**Published:** 2026-03-14

**Authors:** Limoran Tang, Yun Xu, Hui Zhao

**Affiliations:** a Department of Neurology, Nanjing Drum Tower Hospital, Affiliated Hospital of Medical School, Nanjing University, Nanjing 210008, China; b Department of Neurology, Nanjing Drum Tower Hospital, Clinical College of Nanjing University, Nanjing 210008, China; c Department of Neurology, Nanjing Drum Tower Hospital, State Key Laboratory of Pharmaceutical Biotechnology and Institute of Translational Medicine for Brain Critical Diseases, Nanjing University, Nanjing 210008, China; d Department of Radiology, Affiliated Drum Tower Hospital of Nanjing University Medical School, Nanjing 210008, China

Recently, I read with great interest the Appropriate Use Recommendations (AUR) for donanemab presented by Rabinovici et al. in the *Journal of Prevention of Alzheimer's Disease* (2025;12:100,150) [1]. The AUR provide detailed guidance on patient selection, ARIA monitoring, and treatment discontinuation following amyloid clearance. We report a real-world case that closely aligns with these recommendations and demonstrates synchronized normalization of multimodal biomarkers accompanied by clinically meaningful cognitive improvement after donanemab therapy.

A 62-year-old right-handed woman, with junior high school education, presented in April 2025 with a 3-year history of progressive memory impairment, predominantly affecting recent events and conversations. She had well-controlled hypertension and hyperlipidemia, with no stroke history or family dementia. Neurological examination was unremarkable. Cognitive assessment revealed a Mini-Mental State Examination (MMSE) score of 27/30, Montreal Cognitive Assessment (MoCA) score of 18/30, and Clinical Dementia Rating (CDR) score of 0.5, consistent with multi-domain mild cognitive impairment (MCI). Brain magnetic resonance imaging (MRI) revealed mild global cortical atrophy (GCA 1) and Fazekas grade 1 white-matter hyperintensities, with preserved hippocampal volumes.

An ¹⁸F-Florbetapir (AV-45) Aβ positron emission tomography (PET) revealed diffuse cortical amyloid retention, with regional SUVRs of 1.52 in the frontal, 1.58 in the parietal, 1.44 in the temporal, 1.34 in the occipital, and 1.50 in the posterior cingulate cortices. The mean whole-cortex SUVR was 1.48, corresponding to a Centiloid value of 56.98 (Fig. 1A). Tau PET using ¹⁸F-Florzolotau (APN-1607) showed widespread neocortical tau accumulation consistent with Stage C disease (Fig. 1C). Plasma biomarkers, quantified using a surface-engineered microfluidic immunoassay platform, revealed elevated P-Tau217 (0.88 pg/mL) and P-Tau181 (32.95 pg/mL), an increased P-Tau181/Aβ42 ratio (1.23), and a reduced Aβ42/Aβ40 ratio (0.05) [2]. APOE genotyping identified ε3/ε4 status. Based on clinical and biomarker findings, the patient was diagnosed with early symptomatic AD (Clinical Stage 3) meeting the AUR eligibility criteria for donanemab therapy [3].

**Fig. 1:**
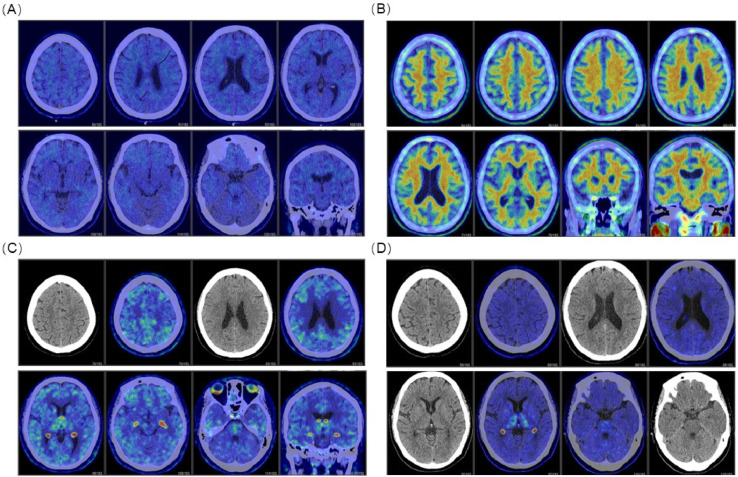
Longitudinal changes in amyloid and tau PET imaging before and after donanemab treatment. (A) Baseline ¹⁸F-Florbetapir Aβ PET obtained in May 2025 showing diffuse cortical tracer uptake. (B) Follow-up Aβ PET performed in November 2025 demonstrating marked reduction and normalization of amyloid burden. (C) Baseline ¹⁸F-Florzolotau tau PET obtained in May 2025 showing widespread neocortical tau deposition consistent with Stage C disease. (D) Follow-up tau PET performed in December 2025 demonstrating near-complete normalization of tracer distribution.

The patient initiated intravenous donanemab therapy in May 2025 per standard protocol, which was well-tolerated with no amyloid-related imaging abnormalities (ARIA) on surveillance MRI. After six months, cognitive performance improved markedly: MMSE 29/30, MoCA 27/30, and CDR 0. Repeat Aβ PET converted to negative, with a Centiloid value of −17.32, accompanied by a ∼20 % reduction in whole-cortex UVR (from 1.48 to 1.18) and concordant regional decreases across all cortical areas (Fig. 1B). Follow-up tau PET similarly showed marked normalization of tracer distribution, with only nonspecific background activity remaining (Fig. 1D). Plasma biomarkers normalized concurrently: P-Tau217 decreased to 0.10 pg/mL, P-Tau181 to 0.86 pg/mL, the P-Tau181/Aβ42 ratio to 0.03, and the Aβ42/Aβ40 ratio increased to 6.32. Structural MRI remained stable without progression of atrophy.

This case illustrates a coherent, multi-level biological response to amyloid-targeted therapy in early AD. The rapid conversion of amyloid PET from positive to negative is consistent with the plaque-clearing efficacy demonstrated in randomized trials [4]. Importantly, amyloid removal was accompanied by substantial attenuation of tau PET signal, despite donanemab not directly targeting tau protein [4]. This temporal coupling supports the mechanistic framework in which upstream amyloid accumulation facilitates downstream tau propagation, and suggests that effective early amyloid clearance may interrupt this cascade before tau pathology becomes self-sustaining. The observation that tau PET signal decreased rather than merely stabilized raises the possibility that, at least in selected early-stage patients, a degree of downstream pathological reversibility may exist. This concept aligns with the AUR’s emphasis on early intervention [1].

The parallel normalization of plasma biomarkers further reinforces the biological coherence of the response. Convergent changes across PET imaging, blood-based markers, and cognition reduce the likelihood that these findings reflect isolated measurement variability. Moreover, the close alignment between plasma P-Tau dynamics and imaging outcomes highlights the potential of blood-based biomarkers as practical tools for monitoring treatment response in routine clinical settings, particularly when repeated PET imaging is limited by cost or accessibility.

Although practice effects cannot be entirely excluded, the magnitude and multidimensional nature of the cognitive improvement supports a treatment-associated effect. The absence of ARIA in this APOE ε3/ε4 carrier further underscores that the risk of donanemab is manageable when administered under structured MRI surveillance in accordance with the AUR [1].

In summary, this real-world observation suggests that, in carefully selected early AD patients, donanemab may induce synchronized improvements across amyloid burden, tau pathology, plasma biomarkers, and cognitive function. These findings support early intervention within a potentially modifiable biological window and reinforce the importance of strict adherence to AUR-based patient selection and monitoring strategies.

Written informed consent was obtained from the patient for publication.

## Declaration of generative AI and AI-assisted technologies in the manuscript preparation process

During the preparation of this work the authors used ChatGPT in order to improve language clarity and readability. After using this tool, the authors reviewed and edited the content as needed and take full responsibility for the content of the published article.

## Source of support

This work was supported by the 10.13039/501100001809National Natural Science Foundation of China (82471454, U25A2064, 82130036), the Nanjing Key Medical Science and Technology Development Project (ZKX23026), the STI2030-Major Projects (2022ZD0211800), and Jiangsu Province Key Medical Discipline (ZDXK202216).

## CRediT authorship contribution statement

**Limoran Tang:** Data curation, Formal analysis, Investigation, Methodology, Visualization, Writing – original draft. **Yun Xu:** Funding acquisition, Supervision. **Hui Zhao:** Conceptualization, Funding acquisition, Writing – review & editing.

## Declaration of competing interests

The authors declare that they have no known competing financial interests or personal relationships that could have appeared to influence the work reported in this paper.
